# Identification and analysis of cuproptosis associated molecular clusters and immunological profiles in atopic dermatitis

**DOI:** 10.3389/fimmu.2025.1545457

**Published:** 2025-06-27

**Authors:** Liangzhe Wang, Bo Wang, Min Peng, Xiaoyan Yang, Sijia Huang, Ruixin Wang, Lin Du, Ruiqian Yao, Wei Wang, Baiping Dong, Yuanjie Zhu

**Affiliations:** ^1^ Department of Dermatology, Naval Medical Center, Naval Medical University, Shanghai, China; ^2^ Department of Ultrasound Diagnosis, Naval Medical Center, Naval Medical University, Shanghai, China; ^3^ School of Medicine, Shanghai University, Shanghai, China; ^4^ Department of Research, Naval Medical Center, Naval Medical University, Shanghai, China

**Keywords:** cuproptosis, atopic dermatitis, differentially expressed genes, immune infiltration, bioinformatics

## Abstract

**Background:**

Atopic dermatitis (AD) is a chronic skin condition marked by persistent itching and dryness. The role of cuproptosis, a novel form of programmed cell death, in AD is not yet understood.

**Methods:**

The GSE107361 dataset was obtained from the Gene Expression Omnibus (GEO) database. Cuproptosis-related genes (CRGs) in AD were identified and analyzed, and immune landscape analysis was performed using ssGSEA. AD was clustered based on CRGs using ConsensusClusterPlus. Weighted gene co-expression network analysis (WGCNA) and differential gene expression analysis were conducted. Hub genes between AD clusters were identified, and both protein-protein interaction (PPI) and drug-gene interaction networks were developed.

**Results:**

Three CRGs (DLD, MTF1, and GLS) were significantly upregulated in the AD group compared to healthy controls. Notably, four core CRGs (LIAS, LIPT1, PDHA1, CDKN2A) distinguished early-onset from adult-onset AD, indicating more active cuproptosis in early-onset AD. CRGs were linked to immune cell infiltration in AD, highlighting differences in immune microenvironments between early- and adult-onset AD. Early-onset AD showed high innate immunity, while adult-onset AD had a mix of innate and type 1 adaptive immunity. CRG expression identified two molecular subtypes with distinct immune infiltration: Cluster 2 (high cuproptosis) had predominant innate immunity, while Cluster 1 (low cuproptosis) had adaptive immunity. Additionally, 102 hub DEGs were identified through WGCNA co-expression network analysis, and 10 hub node genes were identified and potential drugs were explored for the management of AD.

**Conclusions:**

The study provides insights into the roles of cuproptosis-related processes in the pathogenesis and potential treatment of AD. Finding of key hub genes between the 2 distinct immune infiltration subtypes might inform potential therapeutic strategies for AD.

## 1 Background

Atopic dermatitis (AD) is a chronic and refractory inflammatory skin disease, characterized by dry skin and intense pruritus (1). It ranks among the most common dermatological disorders globally, impacting approximately 20% of the world’s population and imposing a substantial societal burden (2). The pathogenesis of AD is intricate and multifactorial, involving complex interactions among genetic predispositions, environmental influences, and immunological mechanisms (3–5). However, the molecular mechanisms of AD are not well understood, hindering its prevention, diagnosis, and treatment. Inflammation, involving various immune cells like macrophages, dendritic cells, mast cells, neutrophils, T cells, and B cells, plays a significant role in AD’s development (6, 7). Furthermore, several studies revealed that early- and adult-onset AD exhibit distinct genetic and immune profiles. For example, adult-onset AD is associated with FLG mutations and Th1 skewing, while early-onset AD shows stronger Th2 polarization. This dichotomy underscores the need for age-stratified analyses.

Copper is an intracellular trace metal essential for numerous biological processes. However, an excess of copper can result in cytotoxicity, although the precise mechanism remains to be fully elucidated (8). *Tsvetkov* et al. were the first to report that cuproptosis represents a copper-dependent and distinct form of cell death, separate from other known types of cell death (9). This form of cell death, which is both copper-dependent and reliant on mitochondrial respiration, occurs through the direct binding of copper to the lipoylated components of the tricarboxylic acid (TCA) cycle. This interaction causes aberrant aggregation of lipoylated proteins and the depletion of iron-sulfur cluster proteins, ultimately resulting in protein toxic stress and the induction of cell death (10, 11).

Recent studies highlight cuproptosis as a key factor in inflammatory disorders. In rheumatoid arthritis, cuproptosis related genes (CRGs) like FDX1 and LIAS increase synovial fibroblast growth and macrophage polarization, worsening joint inflammation (12). In ulcerative colitis, LIPT1 and PDHA1-related cuproptosis disrupts epithelial cells, advancing the disease (13). Notably, in psoriasis, a cutaneous inflammatory disease, cuproptosis causes mitochondrial stress and keratinocyte apoptosis through lipoylation, indicating a common mechanism in immune-related skin conditions (14). However, cuproptosis’s role in AD remains unexamined. Considering AD’s complex immune environment and mitochondrial dysfunction, we hypothesize that CRGs may orchestrate immune cell infiltration and inflammatory responses in AD, contributing to its clinical heterogeneity between early- and adult-onset subtypes. Understanding cuproptosis and CRGs in AD’s immune environment is crucial for exploring its pathogenesis.

The Gene Expression Omnibus (GEO) is a comprehensive database of gene expression datasets related to various diseases (15). These datasets provide crucial information for gaining new insights using different methods. Bioinformatics has become a reliable tool for analyzing existing data (16). In this context, Weighted Gene Co-expression Network Analysis (WGCNA) was used to group selected genes into biological functional modules, which can help explain disease mechanisms (17).

This study utilized the GSE107361 dataset to explore the impact of CRGs on the immune microenvironment in AD, focusing on immune-infiltrating cells, immune-response gene sets, and human leukocyte antigen (HLA) genes. AD samples were categorized into two molecular subtypes, with key molecules identified through the WGCNA co-expression network, highlighting turquoise and yellow modules. A protein-protein interaction network was constructed from 102 hub genes, derived from the intersection of module key genes and 357 differentially expressed genes. Ten hub node genes were identified, suggesting potential therapeutic drugs via DGldb database interactions. This research may provide new diagnostic biomarkers or therapeutic targets for AD.

## 2 Methods

### 2.1 Data collection and preprocessing

The gene expression microarray data GSE107361 were downloaded from the National Center Biotechnology Information Gene Expression Omnibus (NCBI-GEO) database (https://www.ncbi.nlm.nih.gov/geo/, accessed on 15 March 2023). A total of 79 AD patients and 29 adjunct healthy control samples (52 early-onset samples and 56 adult-onset samples) were involved in the present study. During data preprocessing, we retained the samples with both the expression data and the clinical information for analysis, and if there is no probe or multiple genes corresponding to one probe, it is discarded, and if multiple probes correspond to the same gene, the median value is used as the expression amount of the gene. Cuproptosis-related genes were identified from the previous literature (9). A total of 10 genes closely related to cupoptosis were obtained for subsequent analysis, including CDKN2A, FDX1, DLD, DLAT, LIAS, GLS, LIPT1, MTF1, PDHA1, and PDHB.

### 2.2 Chromosome location and protein-protein interaction network of CRGs

Genomic locations of the 10 CRGs in chromosomes were demonstrated using Circos. PPI networks of the CRGs were created from the Search Tool for the Retrieval of Interacting Genes/Proteins (STRING) database, and an interaction score > 0.4 was considered as statistically significant, and hiding individual target protein nodes (18). The data obtained from the String database were input into Cytoscape3.8.0 to visualize the PPI network.

### 2.3 Identification of differentially expressed genes in the CRGs

Based on GSE107361 data, we used Wilcoxon test to analyze the expression of 10 CRGs between AD samples and healthy samples. The DEGs were further identified by the limma package (19) between AD samples and healthy samples. Heat map and box plot were drawn to demonstrate the expressions of DEGs between the two groups. Similarly, the DEGs between AD samples from children and adult patients were identified and illustrated.

### 2.4 Involvement of CRGs in immune regulation of AD

To examine the role of CRGs in the immune regulation of AD, we employed single-sample gene set enrichment analysis (ssGSEA) to quantify immune cell infiltration. Based on GSE107361 data, we conducted a comparative analysis of immune cell infiltration between AD samples and healthy controls and assessed the Spearman correlation between CRGs and immune-infiltrating cells. Furthermore, we validated these findings using the GSE65832 bulk RNA-seq dataset and GSE269981 scRNA-seq dataset comparing AD samples with healthy controls.

Differences in immune response gene set enrichment scores between AD and normal samples were analyzed, and immune response gene sets were downloaded from the immport database (https://www.immport.org/shared/genelists) for a total of 17 immune response gene sets, and spearman correlations between CRGs and immune response gene set enrichment scores were analyzed. Additionally, HLA gene differences between disease and normal samples (PMID: 33724691,17 HLA genes, see supplementary file HLA) were analyzed, and spearman correlations between cuproptosis-related genes and HLA genes were analyzed. Using the same method as above, the immune infiltration, HLA gene difference and immune response gene set among children vs adults patients were analyzed.

### 2.5 Classification of AD samples into two biologically distinct subtypes based on CRGs

To classify AD samples into distinct subtypes based on the expression level of CRGs. The consensus clustering analysis was conducted using the Consensus Cluster Plus package in R software. Optimal clustering K was used and in order to ensure the stability of classification, 1000 repetitions were performed. Principal component analysis (PCA) was used to show the clustering of different CRG-based subtypes. The differential expressions of CRGs in each clusters were analyzed, and the heat map and box plot of cuproptosis-related genes in each clusters were drawn. The differentially expressed genes (DEGs) between cluster 1 and cluster 2 were analyzed by Limma (with log2 fold change (FC) >1 criteria, adjusted P values < 0.05) The differential expressions between groups were identified by limma, with log2 fold change (FC) ≥ 1 and adjusted P values < 0.05 were considered DEGs. This threshold were selected based on established practices for microarray data to balance sensitivity and specificity.

### 2.6 Functional enrichment analysis and drug-gene interaction network

The GO analysis is a useful method for annotating gene and gene product, and identifying characteristic biological meaning of genome and transcriptome (20, 21). The KEGG is a systematic analysis database of gene function, linking genomic information with high-level functional information (22). Based on CRG clusters, the enrichment scores of GO and KEGG were calculated by ssGSEA algorithm, and the enrichment differences of GO and KEGG between two CRG clusters were analyzed and shown in heat map. PPIs with STRING score > 900 were used to construct the interaction network and visualized by cytoscape (23). The Drug-Gene Interaction Database (DGIdb) database serves as a central repository for data on drug-gene interactions and druggability gathered from various sources, providing information on drug-gene interactions and druggable genes (24). The drug-gene interaction network was explored by the DGIdb after excluding all non-specific drugs that targeted > 10 genes from the analysis.

### 2.7 Statistical analysis

All statistical analyses were conducted in R (version 4.3.0) and SPSS (version 27.0) software. For the comparison of continuous variables between two groups, the Mann-Whitney U test (the Wilcoxon rank-sum test) was used, as the data did not meet the assumptions of normality required for parametric tests. The significance levels of p-values were denoted as follows: NS (not significant, p > 0.05), * (0.01 < p ≤ 0.05), ** (0.001 < p ≤ 0.01), *** (0.0001 < p ≤ 0.001), and **** (p ≤ 0.0001). Correlation analyses were performed using the Spearman correlation test, which is suitable for non-parametric data. To control for the false discovery rate (FDR) in multiple comparisons, the Benjamini-Hochberg (BH) correction was applied to adjust p-values where appropriate. Adjusted p-values are reported for all analyses involving multiple comparisons.

## 3 Results

### 3.1 Differential expression analysis of CRGs between AD and healthy samples

**Figure 1:**
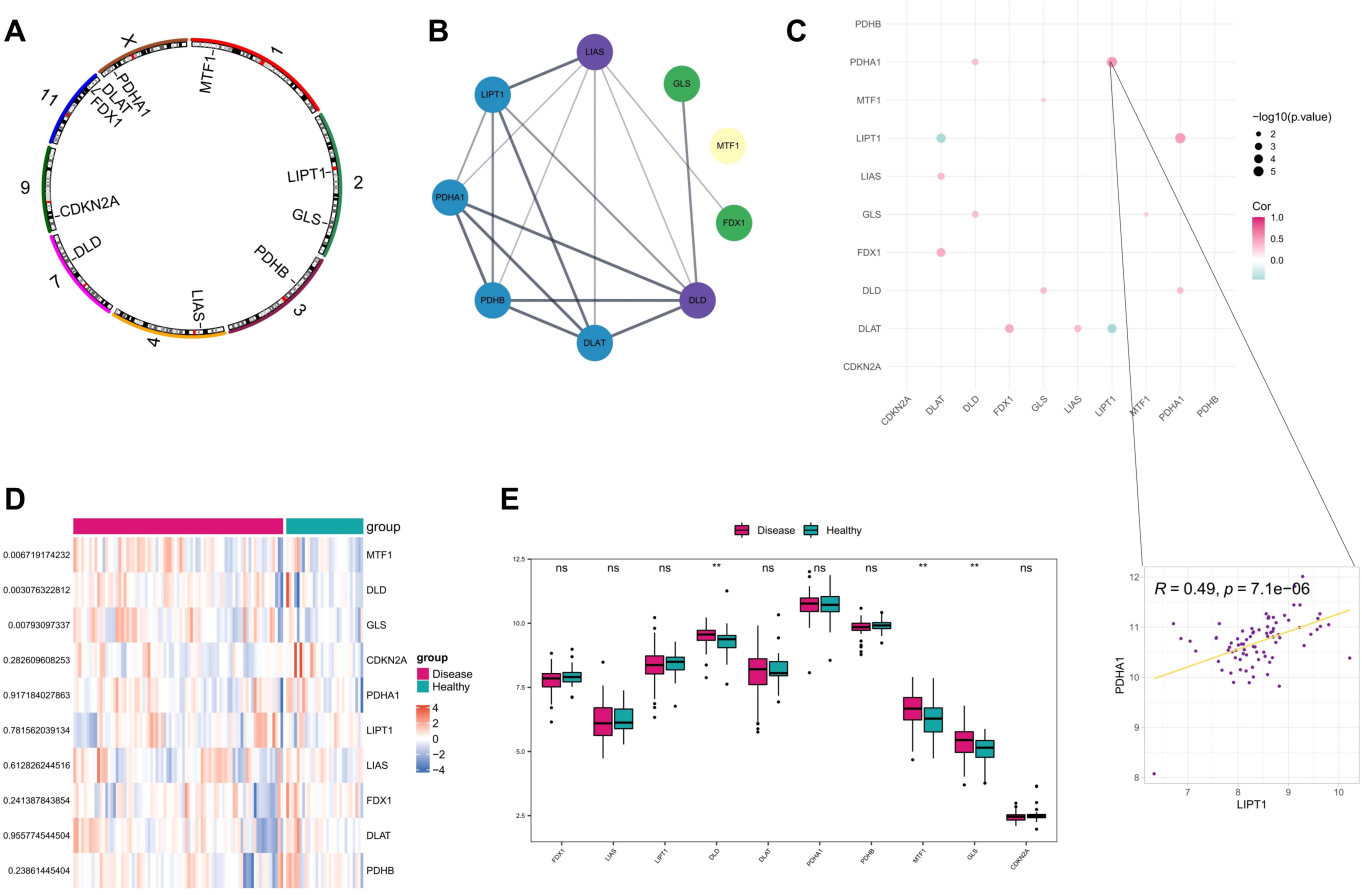
Correlation analysis of AD and CRGs. **(A)** Position of the 10 CRGs on the chromosome. **(B)** Protein interaction network between CRGs; **(C)** Spearman’s correlation between CRGs differentially expressed in disease **(D)** Heat map of 10 CRGs in AD and healthy control samples. **(E)** Box plot demonstrating expressions of 10 CRGs in AD and healthy control samples. **P<0.01, ns, no significance.

As indicated previously, 10 genes (FDX1, LIAS, LIPT1, DLD, DLAT, PDHA1, PDHB, MTF1, GLS, and CDKN2A) were demonstrated to be associated with cuproptosis. First, we analyzed the location of the 10 CRGs on the chromosome (**Figure 1A**), and a PPI network diagram between CRGs was plotted showing the interaction relationship between them (**Figure 1B**). Analysis of the expression correlations between cuproptosis-related genes revealed that LIPT1 and PDHA1 were most correlated among CRGs in AD samples (**Figure 1C**). Then, in order to confirm the involvement of these CRGs in AD, we compared the expression patterns of these 10 CRGs between AD and healthy samples downloaded from GEO database (GSE107361 dataset). Consequently, 3 genes (DLD, MTF1, and GLS) were identified as differentially expressed CRGs between AD and healthy samples, and all of them were upregulated in AD samples (**Figures 1D, E**).

**Figure 2:**
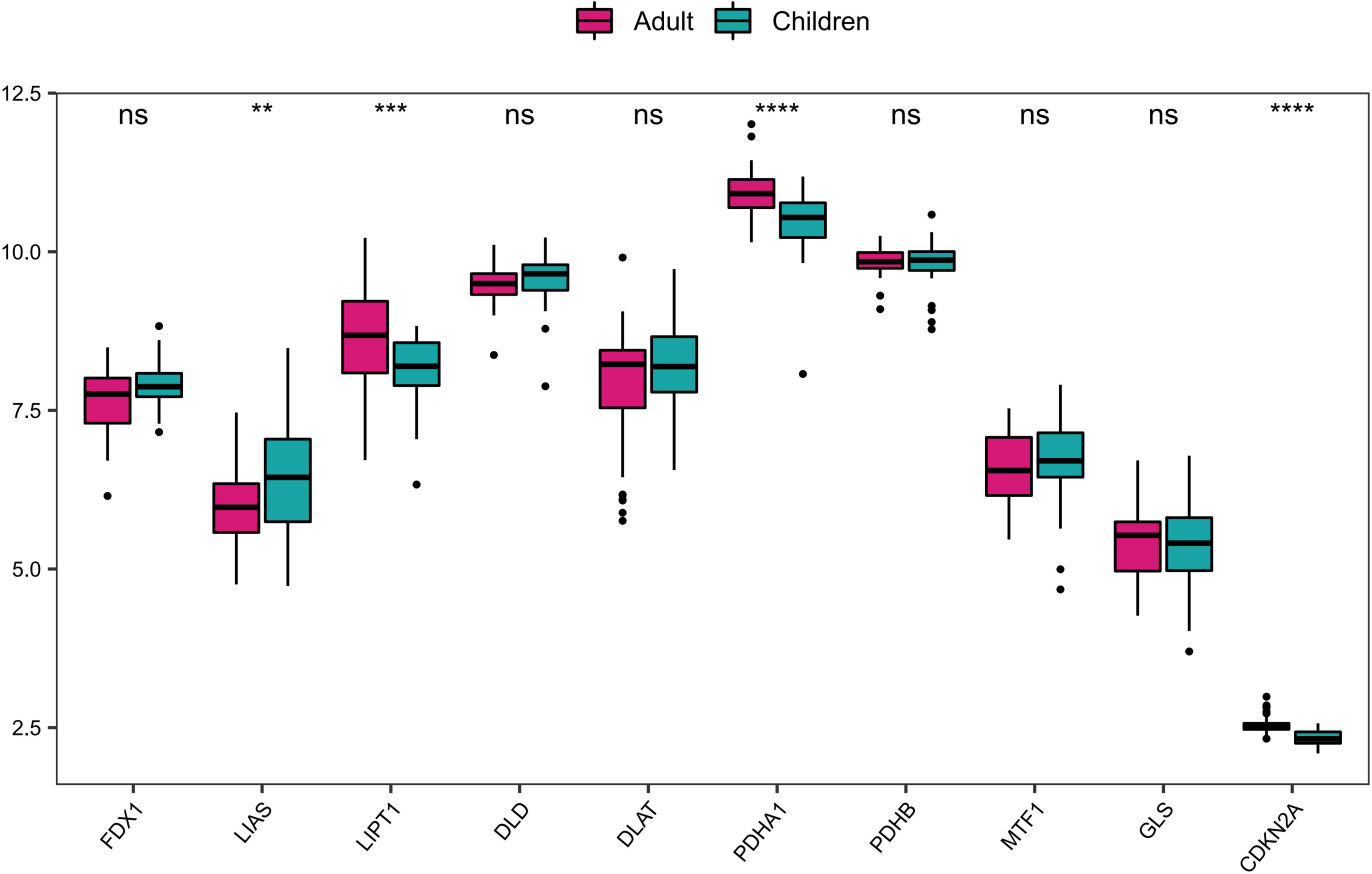
The differential expression of CRGs between early-onset and adult-onset AD. ****P< 0.0001, ***P< 0.001, **P< 0.01, ns, no significance.

Furthermore, based on the expression data of GSE107361 dataset, we used Wilcoxon to analyze the differential expression of CRGs between the samples from early-onset and adult-onset AD. The results showed that LIAS, LIPT1, PDHA1, CDKN2A exhibited significantly differential expression between early-onset and adult-onset AD (**Figure 2**). Among these differentially expressed genes, LIPT1, PDHA1, and CDKN2A were upregulated, while LIAS was downregulated in adult-onset AD samples. These genes are closely related to the condition of immune-infiltrating cells, suggesting the different immune microenvironment between early- and adult-onset AD.

### 3.2 CRGs are involved in the immune regulation of AD

**Figure 3:**
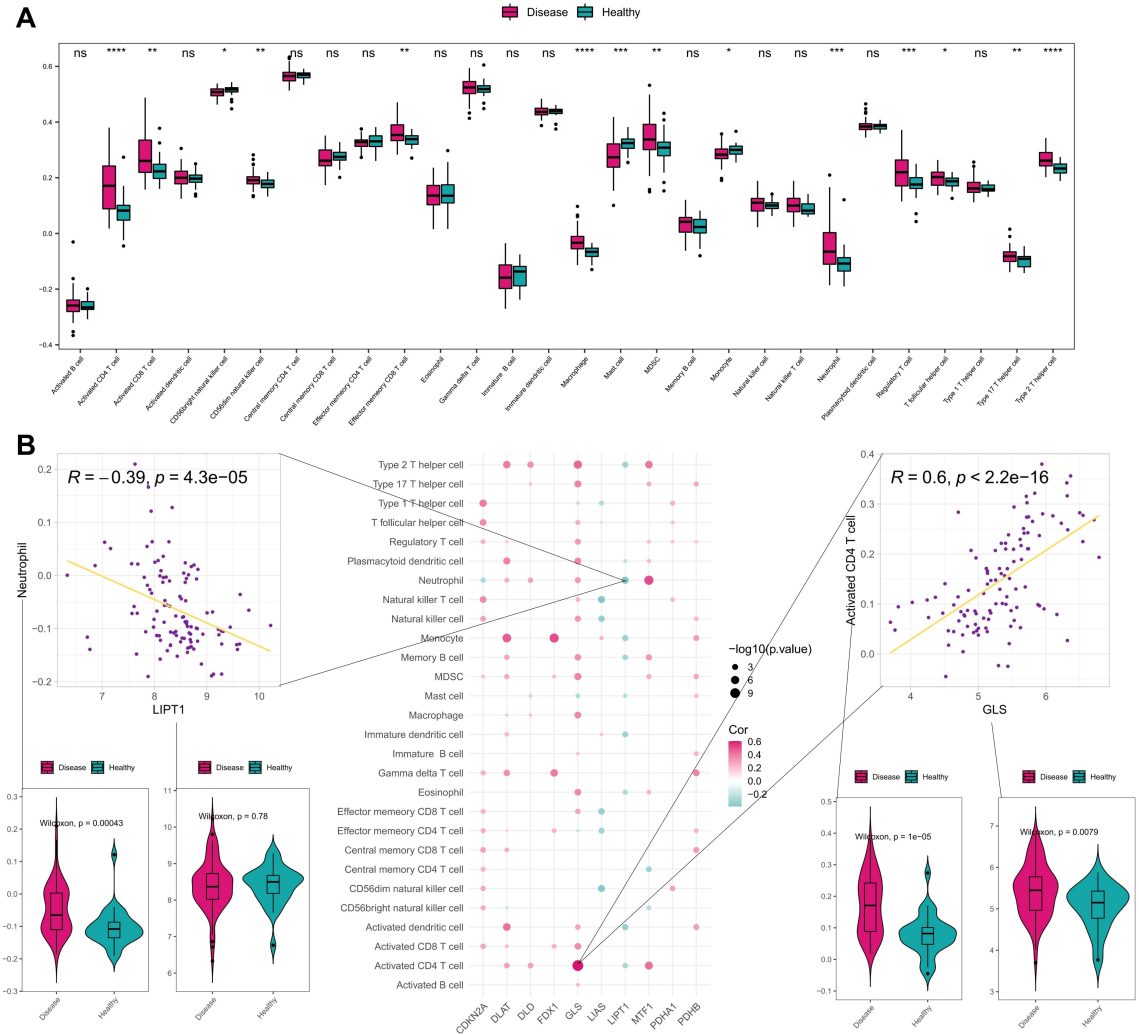
Immune correlation analysis of CRGs in AD and normal samples. **(A)** Differences of various immune cell infiltration scores between disease and normal samples. **(B)** Spearman correlation between CRGs and immune cell infiltration scores, showing scatter plots of the largest positive and negative correlations between them and box plots of the differences between positive and negative correlated immune cells and genes. ****P< 0.0001, ***P< 0.001, **P< 0.01, *P< 0.05, ns, no significance.

To explore the correlation between the development of AD with immune status, the infiltration of immune cells was analyzed between different samples using ssGSEA. Box plots of the immune cell infiltration scores between AD samples and healthy controls were shown in **Figure 3A**, and the infiltration scores of activated CD4 T cell, activated CD8 T cell, effector memory CD8 T cell, macrophage, neutrophil, regulatory T cell, type 17 T helper cell and type 2 T helper cell were significantly higher in disease group than that in healthy controls. This suggests a close association between the development of AD and the immune system.

Furthermore, we validated the ssGSEA results using the GSE65832 bulk RNA-seq dataset and GSE269981 scRNA-seq dataset (AD vs. normal controls). The analysis of bulk RNA-seq dataset (**Supplementary Figure S1**) revealed that the infiltration scores of activated CD4 T cell, effector memory CD8 T cell, neutrophil, myeloid-derived suppressor cell, and type 17 T helper cell were significantly higher in disease group than that in healthy controls, which was consistent with the ssGSEA results, underscoring the reproducibility of our findings. We annotated the immune cells in the scRNA-seq dataset and compared the frequency of immune cells between AD samples and the healthy samples. As shown in the UMAP plots (**Supplementary Figure S2A**), the frequency of immune cells (T cells, B cells, neutrophils, NK cells) in the disease samples is significantly elevated than the healthy samples, validating the increased ssGSEA scores. In addition, Bar plots (**Supplementary Figure S2B**) indicated that AD lesions exhibited higher CD4 T cells, B cells, neutrophils and dendritic cells compared to healthy controls, aligning with ssGSEA results.

The correlation between CRGs and immune cells infiltration were further investigated, and most of CRGs exhibited positive correlation with immune cells. Among them, there was the most significant positive correlation between GLS gene expression and activated CD4 T cell as well as a significant negative correlation between LIPT1 gene expression and neutrophil (**Figure 3B**). These findings suggest that CRGs may regulate immune infiltration in AD.

**Figure 4:**
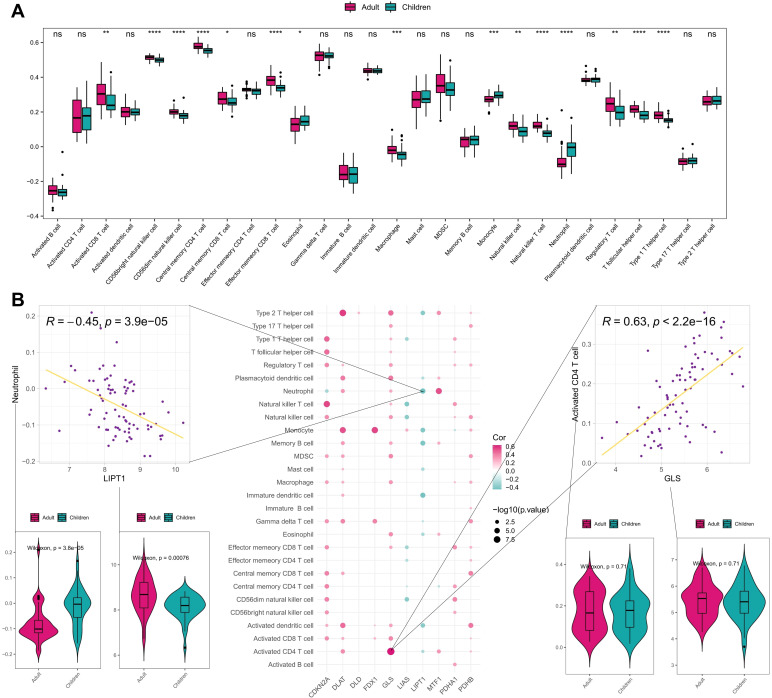
Immune correlation analysis of CRGs in early- and adult-onset AD samples. **(A)** Differences of various immune cell infiltration scores between early- and adult-onset AD samples. **(B)** Spearman correlation between CRGs and immune cell infiltration scores, showing scatter plots of the largest positive and negative correlations between them and box plots of the differences between positive and negative correlated immune cells and genes. ****P< 0.0001, ***P< 0.001, **P< 0.01, *P< 0.05, ns, no significance.

Additionally, we compared the difference of immune cells infiltration scores between early-onset and adult-onset AD samples through ssGSEA. As shown in **Figure 4**, the infiltration scores of activated CD8 T cell, CD56bright natural killer cell, CD56dim natural killer cell, central memory CD4 T cell, effector memory CD8 T cell, macrophage, monocyte, natural killer T cell, neutrophil, regulatory T cell, T follicular helper cell, and type 1 T helper cell, are significantly different between early- and adult-onset AD samples. This suggests that adult-onset AD and early-onset AD may have different immune infiltration characteristics and regulatory mechanisms. The correlation between CRGs and immune cells infiltration in AD samples were further analyzed, and most of CRGs exhibited positive correlation with immune cells infiltration. Consistent with the above results, there was a significant positive correlation between GLS gene expression and activated CD4 T cell as well as a significant negative correlation between LIPT1 gene expression and neutrophil (**Figure 4**). Notably, we found a more extensive enrichment of innate immune cell driven by neutrophil, monocyte, eosinophil in early-onset AD group, while the adult-onset AD group exhibited a more complex immune status combining innate immunity with type 1 adaptive immunity.

### 3.3 Identification of two AD subtypes based on CRG

**Figure 5:**
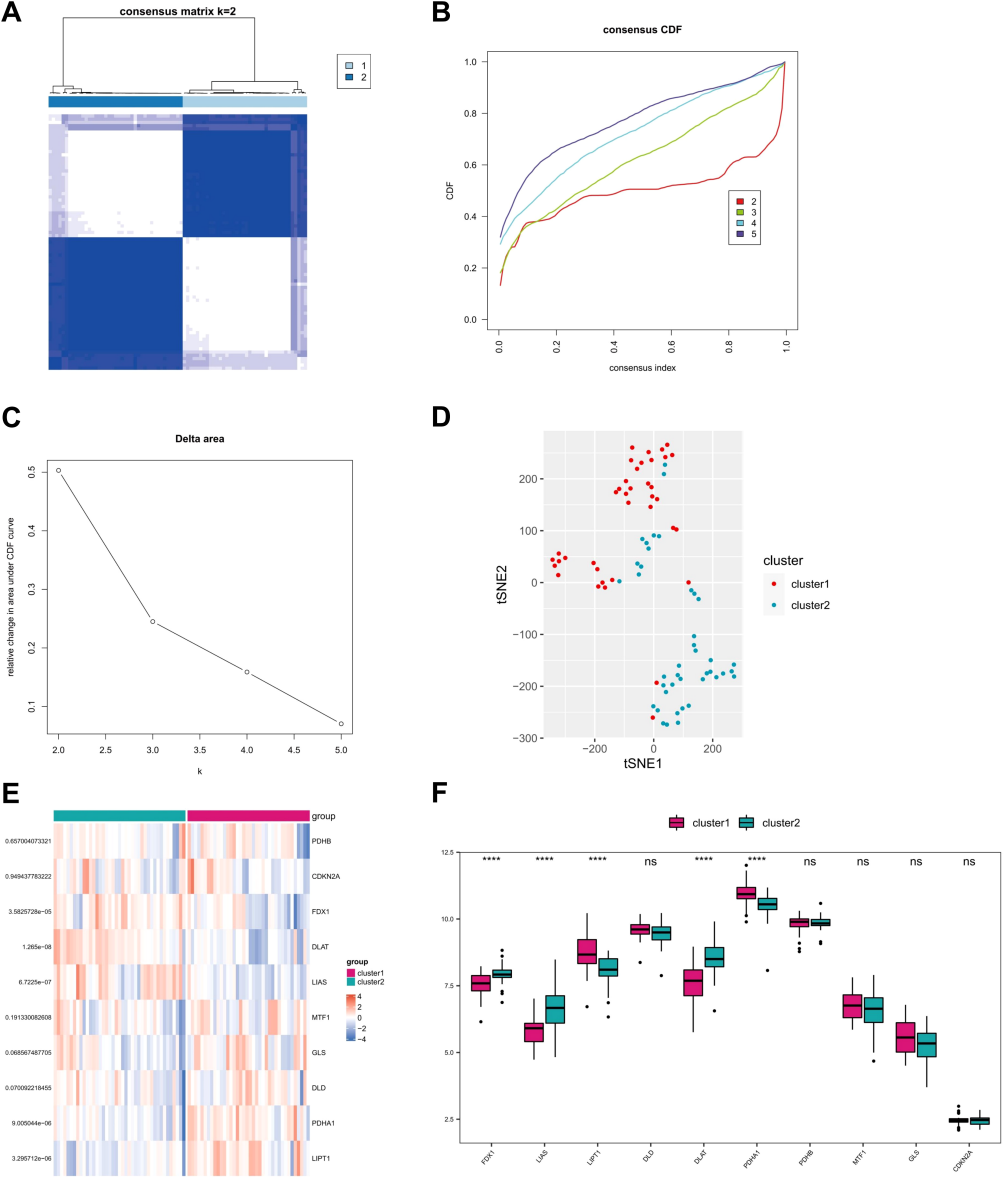
Identification of molecular clusters of CRGs in AD samples. **(A)** Consensus clustering matrix at k=2. **(B, C)** Representative cumulative distribution function (CDF) curves, CDF incremental area curves. **(D)** Principal component analysis (PCA) based on two clusters. **(E, F)** Heat map and box plots illustrating expressions of 10 CRGs in each clusters. ****P<0.0001, ns, no significance.

The consensus clustering analysis was performed using the Consensus Cluster Plus package in R software. Based on the expression profile of 10 CRGs in GSE107361 dataset, the optimal clustering stability was chosen when k = 2 (**Figure 5A**). The curve of CDF values in the range of 0.1-0.9 became nearly smooth when k = 2 (**Figures 5B, C**). Consequently, the 79 AD patients downloaded from GEO database were divided into two groups. Principal component analysis (PCA) demonstrated a good distinction of the two clusters (**Figure 5D**). Subsequently, we conducted the differential expression analysis of CRGs between cluster 1 and cluster 2, and three CRGs (FDX1, LIAS, and DLAT) were found to be higher in cluster 2 than cluster 1, while 2 CRGs (LIPT1, PDHA1) were found to be downregulated in cluster 2 (**Figures 5E, F**). Considering that FDX1 can directly regulate the lipoylation of DLAT through its interaction with LIAS and lead to cuprotosis, cluster 2 with high level of FDX1-LIAS-DLAT exhibited an active cuprotosis status while cluster 1 exhibited a relatively low level of cuprotosis.

**Figure 6:**
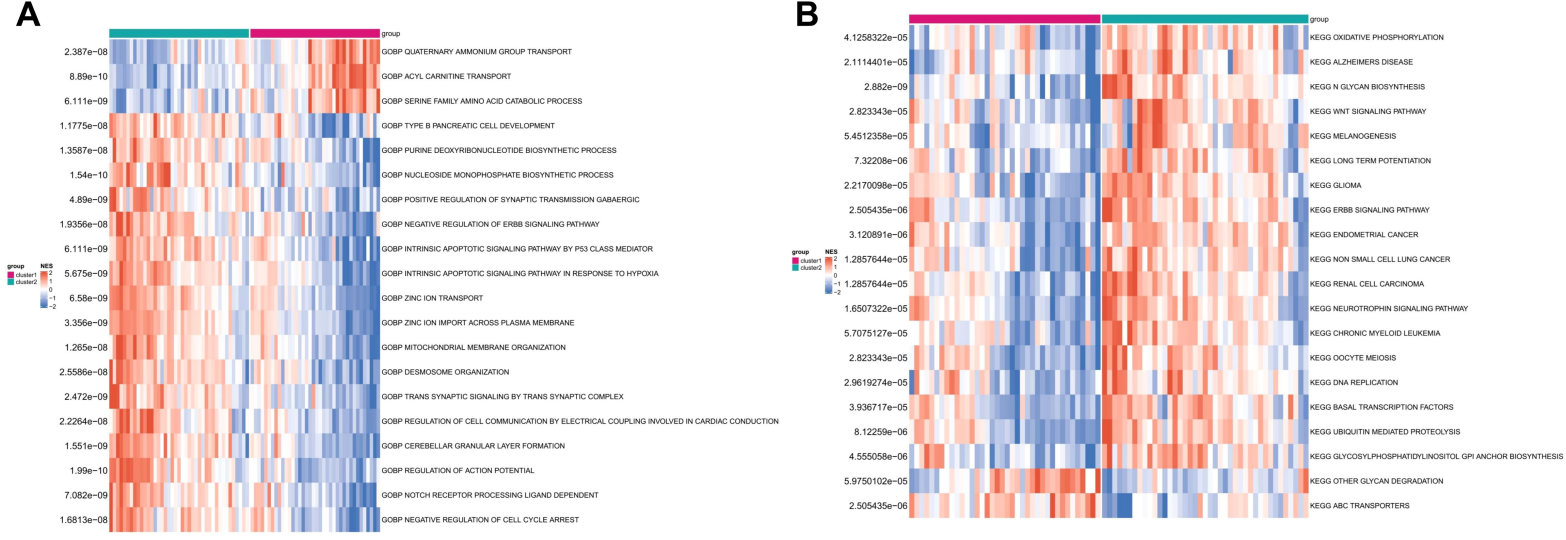
The enrichment difference of GO pathway **(A)** and KEGG pathway **(B)** between the two CRG clusters.

Based on cuproptosis subtype grouping, the enrichment scores of GO and KEGG pathways in different CRG clusters were calculated by ssGSEA algorithm, and the differences of enrichment scores of GO and KEGG pathways between the two clusters were analyzed and shown in heat map (**Figures 6A, B**). It can be seen that there were significant differences in the enrichment scores of GO and KEGG pathways between the two clusters.

### 3.4 Immune infiltration characteristics of CRG based clusters

**Figure 7:**
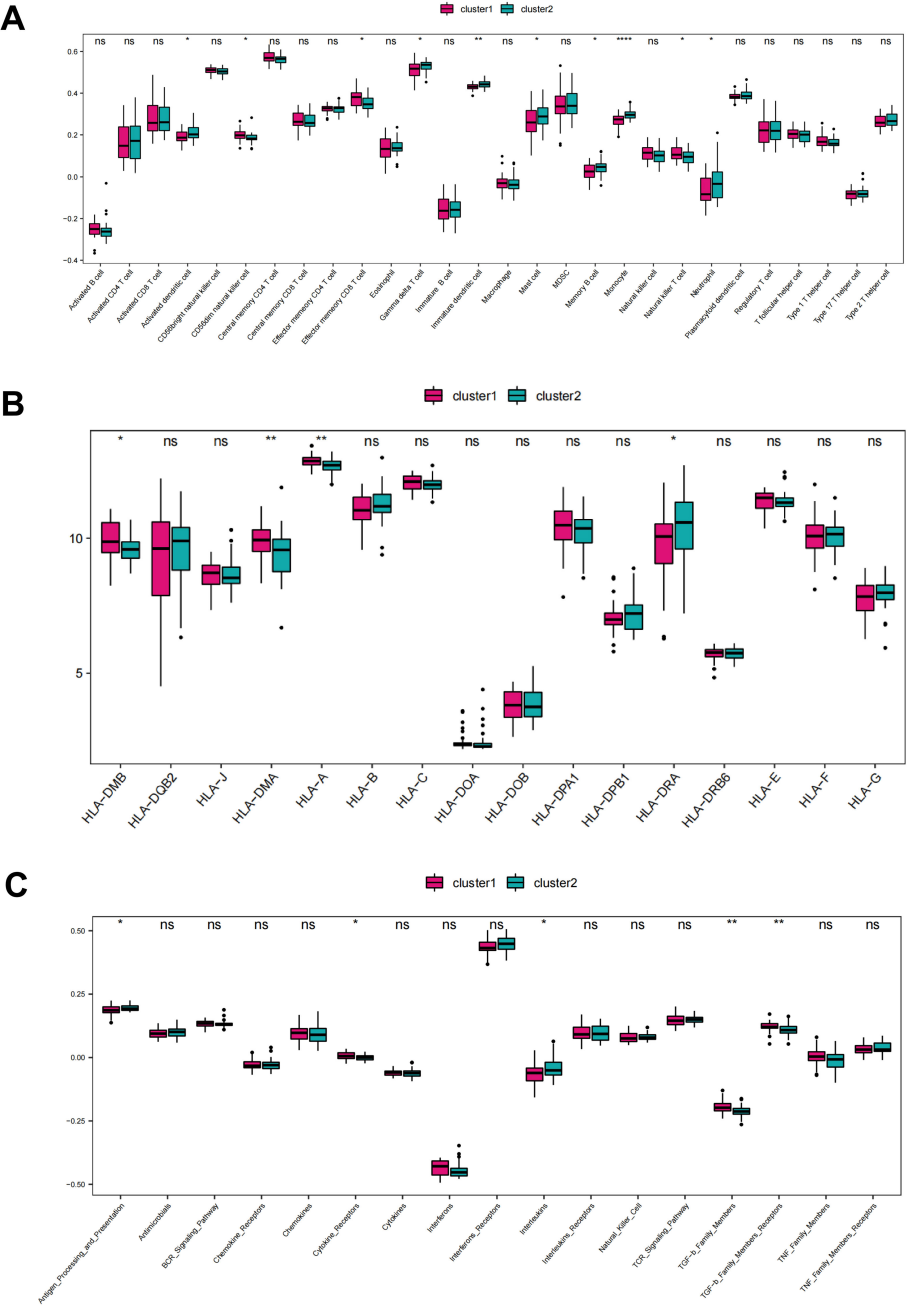
Different immune infiltration characteristics among CRG cluster 1 and 2. **(A)** Box plot showing immune cell infiltration levels in two CRG clusters. **(B)** Box plot demonstrating HLA expressions in two CRG clusters. **(C)** Box plot illustrating immune reaction in two CRG clusters. ns: no significance; *P < 0.05; **P<0.01; ****P<0.0001, calculated by ssGSEA algorithm.

To investigate the differences in immune landscape between the two clusters, the immune infiltrations of cluster 1 and cluster 2 were quantified and analyzed. The results demonstrated that there were significant differences of infiltration scores in activated dendritic cell, CD56dim natural killer cell, effector memory CD8 T cell, gamma delta T cell, immature dendritic cell, mast cell, monocyte, natural killer T cell, and neutrophil between the two clusters. Cluster 1 showed higher levels in CD56dim natural killer cell, effector memory CD8 T cell, and natural killer T cell. We term cluster 1 as adaptive-immunity predominant subtype (AI-subtype), since all of these cells showed a function of cytotoxicity, with more perforin and granztmes production or a short half-life and little memory potential, which further regulate the adaptive immunity. While cluster 2, termed as innate-immunity predominant subtype (II-subtype), showed higher levels in activated dendritic cell, immature dendritic cell, gamma delta T cell, mast cell, monocyte, and neutrophil (**Figure 7A**), which were mainly function in innate immunity. The accumulation of immune cells with different functions implies different immune responses, resulting from different stimuli factors. Similarly, there were significant differences of gene expression in HLA-A, HLA-DMB, HLA-DMA, and HLA-DRA between the two clusters, and cluster 1 showed higher expression in HLA-A, HLA-DMB, HLA-DMA than cluster 2 (**Figure 7B**). Additionally, the enrichment scores of cytokine receptors, antigen processing and presentation, were significantly different between CRG-based clusters (**Figure 7C**).

Furthermore, we performed the breakdown of early- vs adult-onset samples in clusters 1 and 2. As shown in **Table 1**, cluster 1 consisted of 68.4% early-onset and 31.6% adult-onset AD samples, while cluster 2 included 36.6% early-onset and 63.4% adult-onset AD samples. There was a significant correlation between CRGs-based subtype (cluster 1 and cluster 2) and age-based subgroup (adult-onset AD and early-onset AD) (P<0.05), which is consistent with the observation that there were similar immune differences in early- vs. adult-onset comparison and cluster 1 vs. 2 comparison points.

**Table 1:** Breakdown of early- vs adult-onset samples in clusters 1 and 2.

CRGs-based subtypeAge-based subgroup	Cluster 1 (38)	Cluster 2 (41)
Adult-Onset	26 (68.4%)	15 (36.6%)
Early-Onset	12 (31.6%)	26 (63.4%)

Statistical Test: Significant association between clusters and age groups (χ^2^ = 6.7822, P<0.05).

### 3.5 Identification of key molecules based on co-expression network

**Figure 8:**
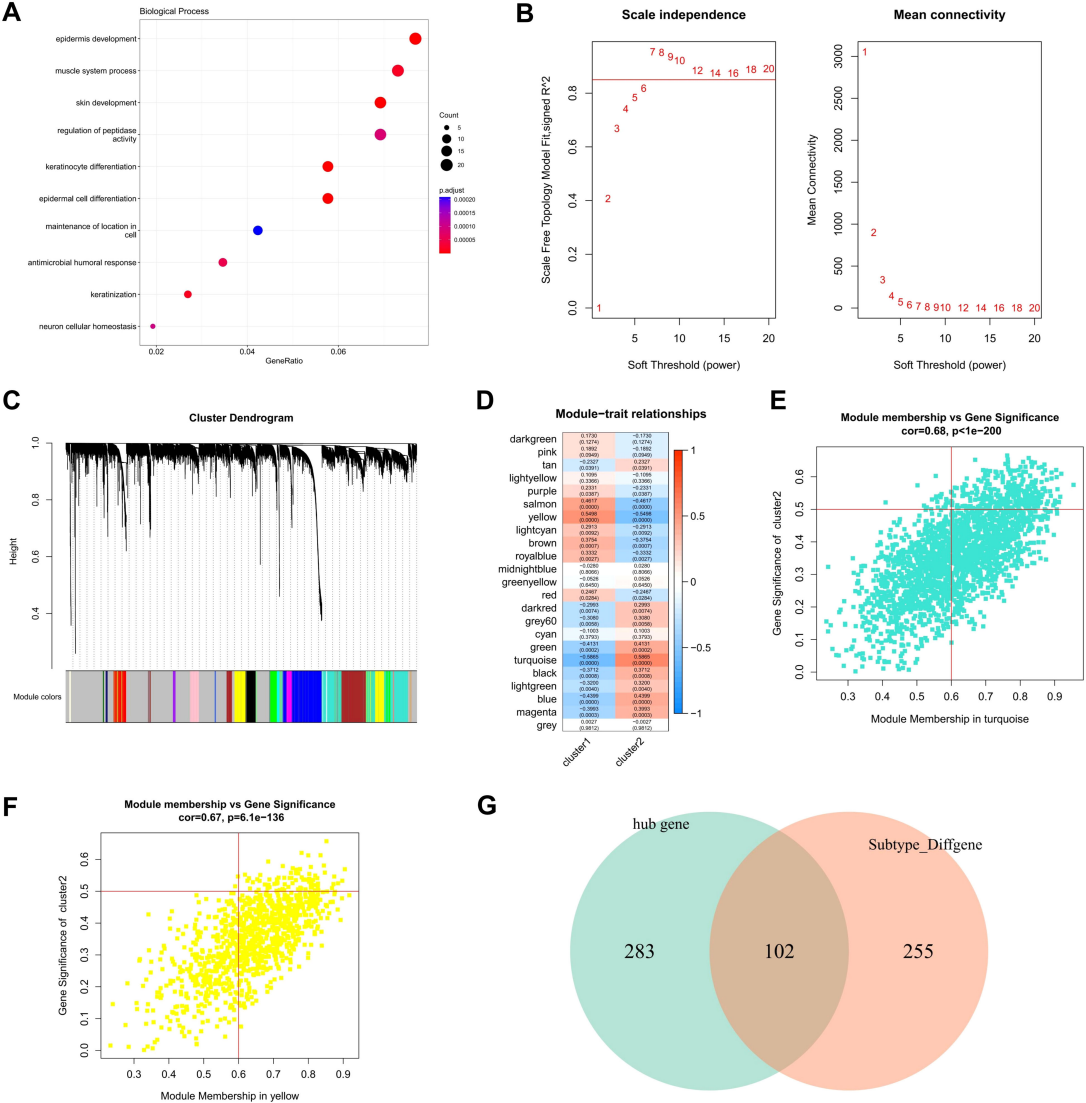
Hub genes screening by WGCNA. **(A)** Biological characteristics of the differentially expressed genes between two clusters were revealed by GO functional enrichment analysis. **(B)** Analysis of network topology for various soft-thresholding powers. **(C)** Gene tree map obtained by hierarchical clustering, with color lines below the tree showing the allocation of modules determined by dynamic tree cutting, which identified 23 modules. **(D)** Heat map of module feature correlation **(E)** Turquoise scatter map of the genes in the module with MM and GS, with GS and MM showing relatively significant correlation. **(F)** yellow scatter map of the genes in the module with MM and GS, with GS and MM showing relatively significant correlation. **(G)** Venn map of 385 hub genes with the 357 DEGs between CRG-based clusters.

Through bioinformatics analysis, a total of 357 differentially expressed genes (DEGs) were found between cluster 1 and cluster 2. To further verify the biological processes related to these DEGs between the two clusters, GO enrichment analysis was performed using the cluster profiler to explore relevant biological process (**Figure 8A**). Several biological processes, such as epidermis development, muscle system process, skin development, regulation of peptidase activity, keratinocyte differentiation, epidermal cell differentiation, maintenance of location in cell, antimicrobial humoral response, keratinization, neuron cellular homeostasis were enriched.

Based on these gene expression profiling, the weighted co-expression network was constructed using WGCNA. The results showed that co-expression network conforms to the scale-free network, that is, the log (K) of the node with K connectivity is negatively correlated with the log (p (K)) of the probability of the node occurrence, and the correlation coefficient is greater than 0.8. To ensure that the network was scale-free network, we chose optimal β = 7 (> 0.85, **Figure 8B**). The next step was to convert the expression matrix to an adjacency matrix, which was then converted to a topological matrix. We used average-linkage hierarchical clustering to cluster genes according to the criteria of mixed dynamic shear trees, and set the minimum number of genes to 30 per gene network module. After identifying gene modules using the dynamic shear method, we calculated the eigengenes of each module once and then performed cluster analysis on the modules, merging the closer modules into the new module with a set height = 0.25, a total of 23 modules were obtained (**Figure 8C**).

Pearson correlation coefficients were calculated for the ME of each module and the phenotypic characteristics of the sample, with higher values representing the module being more important. The rows in **Figure 8D** represent the feature vector genes for each module, and the columns represent the sample phenotypic characteristics, and the correlation coefficients decrease from high to low in descending order from red to blue. The numbers in each box represent the coefficient of correlation between the gene module and the phenotypic characteristics of the sample, and the numbers in parentheses indicate the significance of the P value. Turquoise and yellow were chosen as the key modules. According to the criterion that is module membership (MM) > 0.6 and gene significance (GS) > 0.5, a total of 385 module hub genes (**Figures 8E, F**) were obtained. Finally, a total of 102 hub DEGs were obtained by crossing the 385 hub genes with the 357 DEGs between CRG-based clusters (**Figure 8G**).

### 3.6 Potential therapeutic strategies

**Figure 9:**
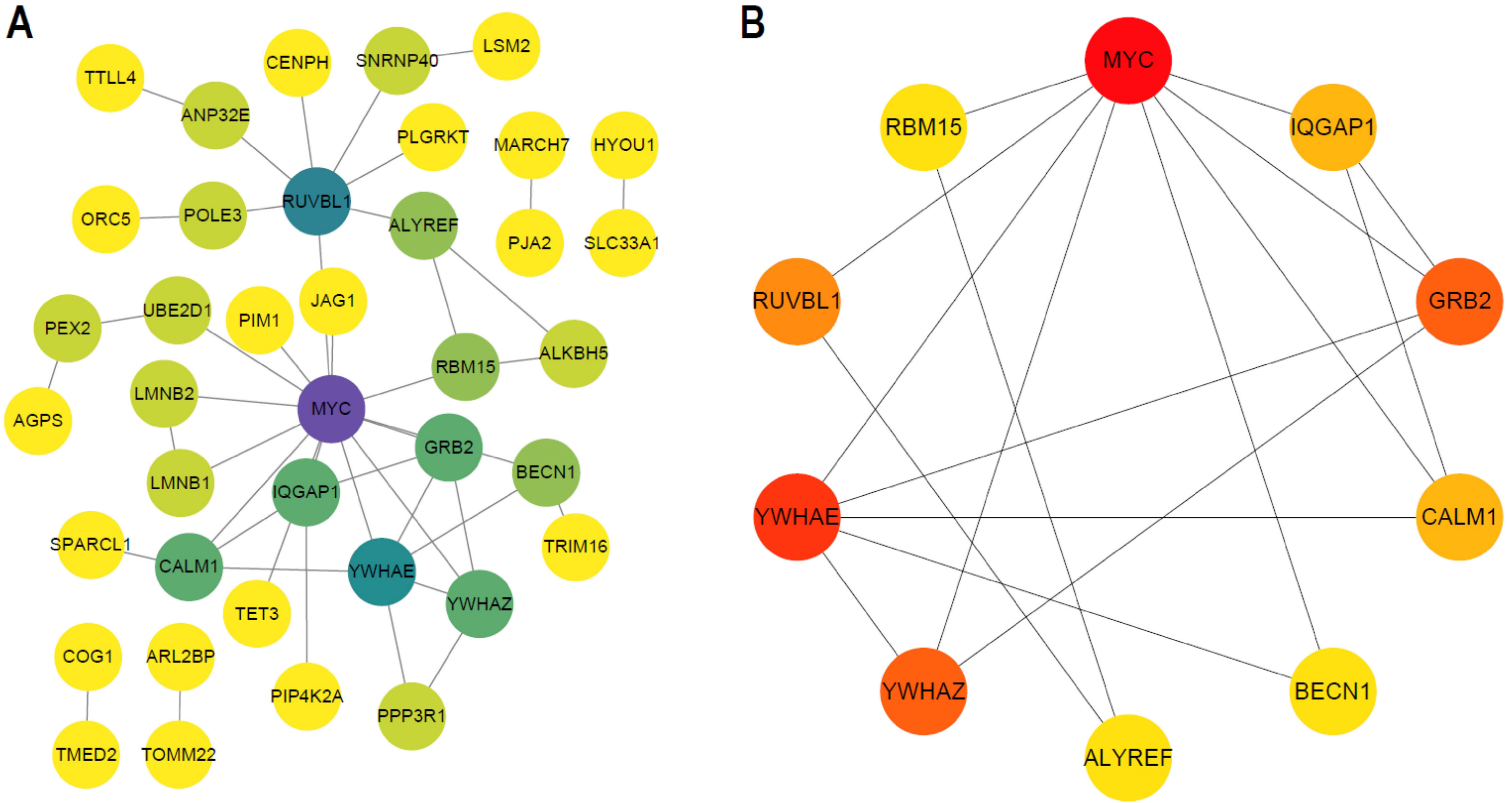
Key hub genes screening. **(A)** PPI network map of hub DEG genes. **(B)** PPI network map of TOP10 genes using MCC methods of Cytoscape.

**Figure 10:**
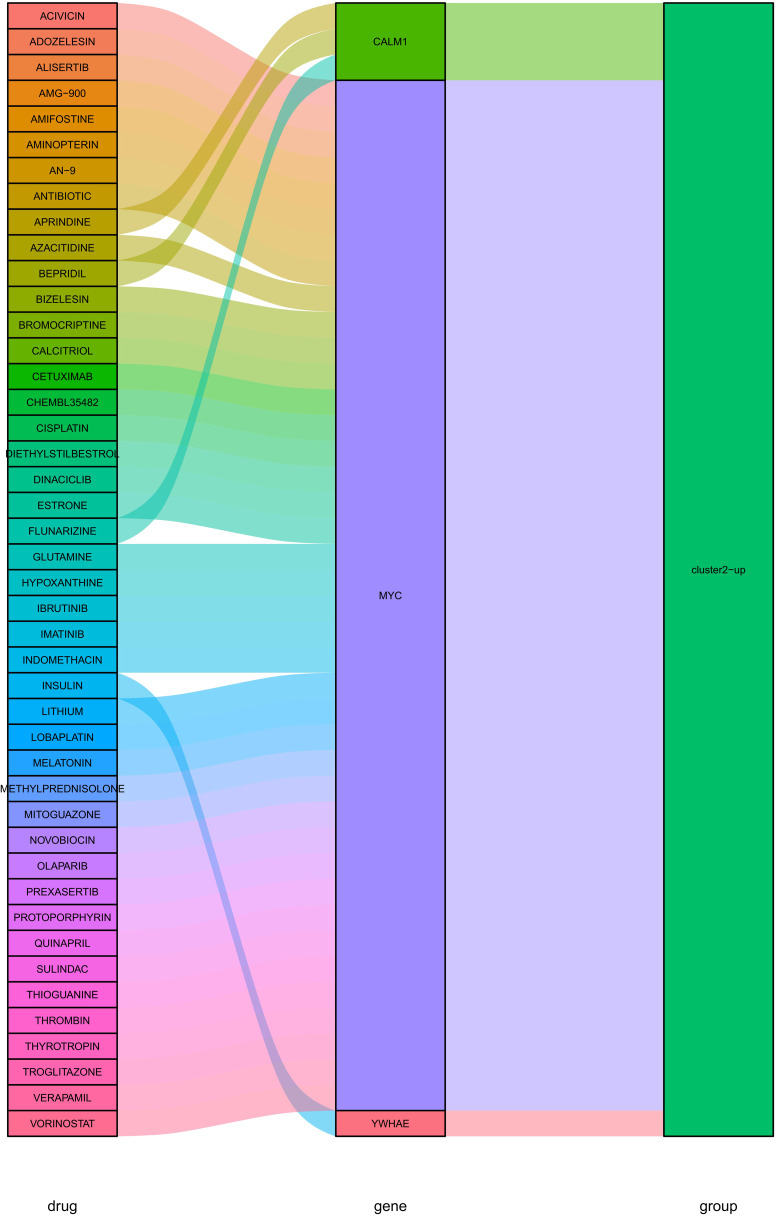
Interactions between hub key genes with drugs identified in DGlDB database.

PPI network was constructed based on the above identified hub DEGs (based on STRING database) (**Figure 9A**). Hub nodes in PPI network were investigated using MCC method of Cytoscape, and 10 genes were obtained as the key hub genes, including YWHAE, CALM1, MYC, RBM15, RUVBL1, YWHAZ, ALYREF, BECN1, GRB1, IQGAP1 (**Figure 9B**). Further, the interactions between these key genes with drugs were explored based on the DGIDB database v4.2.0 (https://www.DGIdb.org/), and 3 genes, including YWHAE, CALM1, and MYC, were confirmed that can interact with the specific drugs in the DGlDB database, and these corresponding drugs may be the potential target drugs for addressing AD (**Figure 10**). Future studies should evaluate drug responses in subtype-specific models.

## 4 Discussion

Atopic dermatitis is a refractory inflammatory skin disease manifested as dry skin, intense itching, and intense itch, bringing increasing medical burden worldwide in the past few decades (25). It has multiple risk factors involved in the interaction between genetic susceptibility and various environmental stimuli (26, 27). Due to the unclear etiopathogenesis and complex pathophysiological process of AD, its prevention and treatment remain challengeable. Therefore, a comprehensive understanding of the genetic backgrounds and cutaneous immune microenvironment is essential for the prevention, treatment, and prognosis evaluation of AD.

Cuprotosis is a newly reported cell death form dependent on mitochondrial respiration and TCA cycle (11). It has been widely studied in various tumor and inflammatory disorders since its mechanism first being clarified by Tsvetkov et al. in 2022. Recently, several studies have confirmed that cuprotosis-related genes (CRGs) exhibit critical regulatory effects on the immune microenvironment of a variety of inflammatory disorders, such as rheumatoid arthritis, Crohn’s disease, and ulcerative colitis (12, 13, 28, 29). In psoriasis, as a cutaneous immune-mediated inflammatory disease (cIMID), cuproptosis has been confirmed being associated with mitochondrial metabolism and facilitated through protein lipoylation (14). However, in AD, another well-known cIMID, the specific mechanisms and regulatory roles of CRGs have never been investigated.

In the present study, we conducted the first comprehensive analysis of the differential expression of CRGs between healthy and AD patients. Consequently, 3 out of 10 CRGs, including MTF1, DLD, and GLS, were identified as the differentially expressed genes between AD and healthy samples, and all of them were upregulated in AD samples, suggesting the close association of these CRGs with the development of AD. Previous studies reported that DLD and MTF1 expression positively correlated with activated CD8T cells, activated CD4T cells, eosinophils, type 2 T helper cells, and type 17 T helper cells (30). Notably, these immune cells are all the key drivers in the immune microenvironment of AD (31, 32), further confirming our findings that DLD and MTF1 play a critical role in the development of AD by regulating the immune infiltration.

In addition, as an essential molecule for Th17 cell production (29), the upregulation of GLS expression in AD samples demonstrated the potential roles of Th17 pathway in the pathogenesis of AD. Notably, previous research demonstrated that GLS can also promote the occurrence of inflammatory bowel diseases (IBD) (33), and thus the AD patients may have an increased risk of IBD, which has been confirmed by a study conducted on the risk of inflammatory bowel disease in patients with AD (34). These findings fully indicate that GLS gene can be used as a promising target, which can not only intervene in the Th17 pathway, thereby inhibiting inflammatory response, but also effectively prevent and treat AD patients with IBD.

Similarly, we also explored and identified the differentially expressed CRGs between early-onset and adult-onset AD samples. Among them, the expressions of LIPT1, PDHA1, and CDKN2A were upregulated in adult-onset sample, while the expression of LIAS was upregulated in early-onset sample. A study conducted on ulcerative colitis (UC) revealed that both LIPT1 and PDHA1 are key genes to promote cuproptosis, which can induce the abnormal death of intestinal epithelial cells and lead to the occurrence of UC (28). According to the results in our study, both LIPT1 and PDHA1 were significantly upregulated in adult-onset AD samples. Thus, we deduce that the adult-onset AD patients may have increased risk of UC compared to early-onset AD patients. This speculation was confirmed by a study conducted on the risk of inflammatory bowel disease that adult-onset AD had a 32% increased risk of UC, while early-onset AD did not have increased risk of UC (34). These evidence demonstrate that LIPT1 and PDHA1 may act as the shared targets for the diagnosis, prognosis and treatment of AD and UC.

Several studies have demonstrated that AD is a common disease that is associated with atopic and nonatopic comorbidities, such as s asthma, rhinitis, and food allergy, psychiatric, autoimmune, cardiovascular diseases, and certain cancers (35–38). Considering the critical roles of cuproptosis in these diseases (10, 37, 39, 40), CRGs may provide a novel perspective on the underlying mechanisms of AD comorbidity with these diseases.

Notably, LIAS, also a key gene that promotes cuproptosis, was significantly upregulated in early-onset AD samples. LIAS encoded components of the lipoic acid pathway and synthesized a potent antioxidant termed α-Lipoic acid (LA) in mitochondria and it was found to be associated with oxidative stress and inflammation. Previous studies demonstrated that LIAS expression was highly correlated with the infiltration of immune cells, such as Treg cells, Th1 cells, Th2 cells, NK cells, CD4 T cells, CD8 T cells, neutrophil, macrophages, and et al. (41). The result that LIAS was the only CRGs upregulated in early-onset AD sample suggests the different immune-related characteristics between early-onset and adult-onset AD group. This helps us to recognize the different pathogenesis of adult-onset and early-onset AD and to conduct different treatment strategies.

What’s more, the immune landscape, including immune cell infiltration, immune response and HLA genes, in AD samples was analyzed using ssGSEA. Most of CRGs exhibited positive correlation with enhanced infiltration and activation of immune cells and immune-related signaling. Among them, there was a significant positive correlation between GLS gene expression and activated CD4 T cell as well as a significant negative correlation between LIPT1 gene expression and neutrophil. CD4+ T cells are known as the principal drivers of AD, and neutrophils can produce reactive oxygen species and led to skin barrier damage in AD.

The concept of precision medicine has promoted the clustering of individual subjects (42). In this study, we performed an unsupervised clustering analysis based on CRG expression levels and identified two biological clusters in AD samples. The expressions of CRGs exhibited significant difference between the two clusters. Three differentially expressed CRGs, namely FDX1, LIAS, and DLAT, were expressed at significantly higher levels in cluster 2 than cluster 1 and had higher levels of immune infiltration in activated dendritic cell, gamma delta T cell, immature dendritic cell, mast cell, monocyte, and neutrophil. Similarly, there were significant differences of gene expression in HLA-A, HLA-DMB, HLA-DMA, and HLA-DRA between the two clusters, and cluster 1 showed higher expression in HLA-A, HLA-DMB, and HLA-DMA than cluster 2. Pathway enrichment analysis also revealed a number of immune-related pathways that were significantly activated in cluster 2. Taken together, these findings demonstrated that different cuprotosis activation status exhibit distinct immune infiltration characteristics (innate-immunity predominant or adaptive-immunity predominant). Hence, CRGs may regulate the pathological process of AD by mediating these classical pathways associated with cell death, metabolism, and immune response. Future work should also investigate transcriptional regulators (e.g., NF-κB, HIF-1α) and epigenetic modifiers (e.g., DNA methylation, histone acetylation) that govern CRG expression in AD. Single-cell ATAC-seq or ChIP-seq in CRG-defined subtypes could elucidate regulatory networks linking copper metabolism to immune dysfunction.

We further performed the breakdown of early- versus adult-onset samples in clusters 1 and 2 and identified a significant correlation between CRG-based subtypes and age-based subgroups. The observed similarities between cluster 1 and adult-onset cases, as well as cluster 2 and early-onset cases, suggest that CRG clusters can be approximately inferred from the age of AD patients, potentially facilitating targeted management strategies for AD. Nonetheless, the presence of early-onset AD patients in cluster 1 and adult-onset, including elderly AD patients, in cluster 2 indicates that classification based solely on clinical age is imprecise. Therefore, classifying AD through CRG analysis may offer more effective guidance for clinical treatment and prognosis.

Furthermore, the 10 hub genes identified (e.g., MYC, CALM1, YWHAE) are mechanistically linked to AD pathogenesis. MYC, a master transcriptional regulator, drives Th17 differentiation and IL-17 production—a cytokine pivotal in AD-associated skin inflammation (43, 44). CALM1 modulates calcium signaling, which is critical for epidermal barrier integrity; its dysregulation may exacerbate transepidermal water loss in AD. YWHAE, a 14-3–3 protein, regulates apoptosis and T cell activation, potentially influencing AD’s chronicity (45). Clinically, these genes offer therapeutic potential. For example, Vorinostat—an HDAC inhibitor targeting MYC—suppresses Th17 responses in preclinical models of psoriasis (46, 47), suggesting repurposing potential for AD. Similarly, insulin, which interacts with CALM1, may restore barrier function via calcium homeostasis (48). Future studies should prioritize validating these targets in AD-specific models and clinical trials.

This study is the first to examine the potential roles of cuproptosis and CRGs in AD. Cuproptosis is a unique form of cell death that relies on mitochondrial respiration. Understanding the role of CRGs in the immune microenvironment of AD could shed light on the disease’s development. However, there were still some limitations in our study. First, the predominance of European patients in GSE107361 limits the generalizability of our findings to other ethnic groups. Future studies should validate CRG signatures in diverse cohorts, including Asian and African populations, to account for genetic and environmental heterogeneity. Second, the modest cohort size of GSE107361 (79 AD patients, 29 controls) restricts robust stratification of AD subtypes by clinical severity or comorbidities. Larger, prospectively collected cohorts are needed to validate the prognostic utility of CRG clusters. Third, while bioinformatics approaches robustly identify associations, they cannot establish causality; experimental validation (e.g., CRISPR knockout of DLD or MTF1 in AD models) is essential to confirm mechanistic roles. Lastly, although drug-gene interactions were predicted, their efficacy and safety in AD require rigorous preclinical testing. Addressing these limitations will strengthen the translational relevance of our findings.

## 5 Conclusion

This study is the first to examine CRGs in AD and their impact on the immune microenvironment. It identified three CRGs (DLD, MTF1, GLS) with differential expression between AD and healthy samples, and four CRGs (LIAS, LIPT1, PDHA1, CDKN2A) with significant expression differences between early-onset and adult-onset AD. These genes could be crucial for understanding AD’s immunological mechanisms. AD samples were classified into two molecular subtypes based on cuproptosis, corresponding to adaptive or innate immunity predominance. Using WGCNA and gene-drug interaction analysis, 10 hub genes and potential therapeutic drugs, such as acivicin and lithium, were identified for AD management. All these findings suggest that CRGs may play a pivotal role in AD pathogenesis, and ultimately, the identification of CRGs could be a novel perspective on biological markers of diagnosis and treatment of AD.

## Data Availability

The original contributions presented in the study are included in the article/**Supplementary Material**. Further inquiries can be directed to the corresponding authors.
