# Supplementary figures and images for: Identification and analysis of cuproptosis associated molecular clusters and immunological profiles in atopic dermatitis
Source: Front Immunol. 2025 Jun 27;16:1545457. doi: 10.3389/fimmu.2025.1545457 (PMC12245679; doi:10.3389/fimmu.2025.1545457)

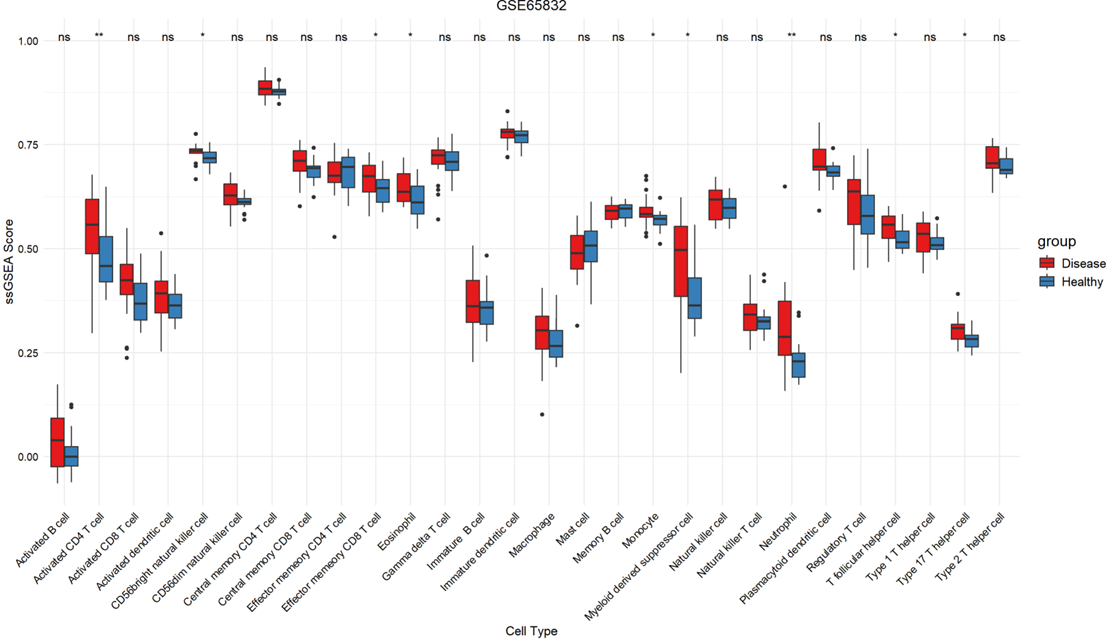

Supplement: Supplementary file 1 [file Image1.tif]

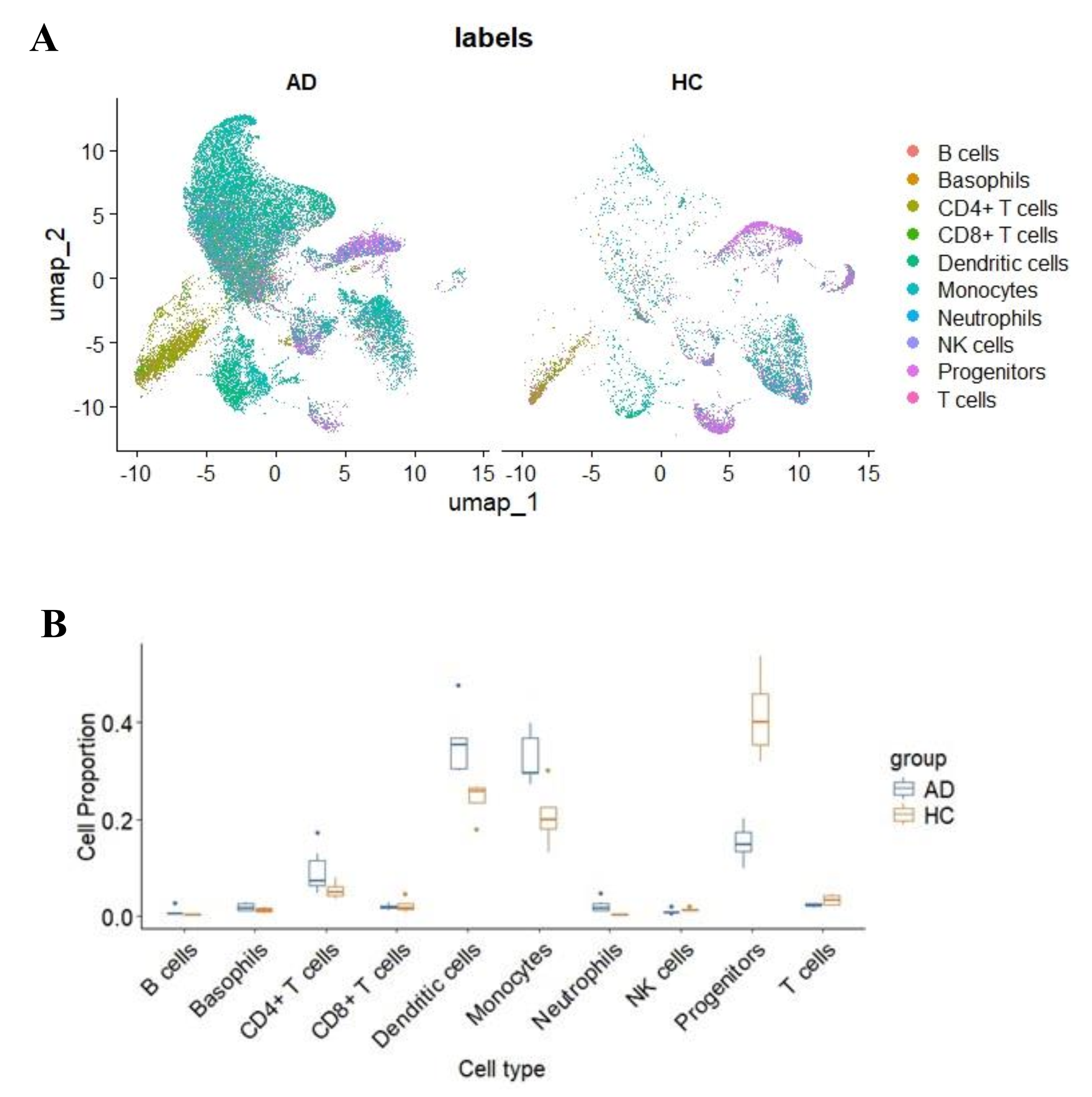

Supplement: Supplementary file 2 [file Image2.tif]

Disease Healthy

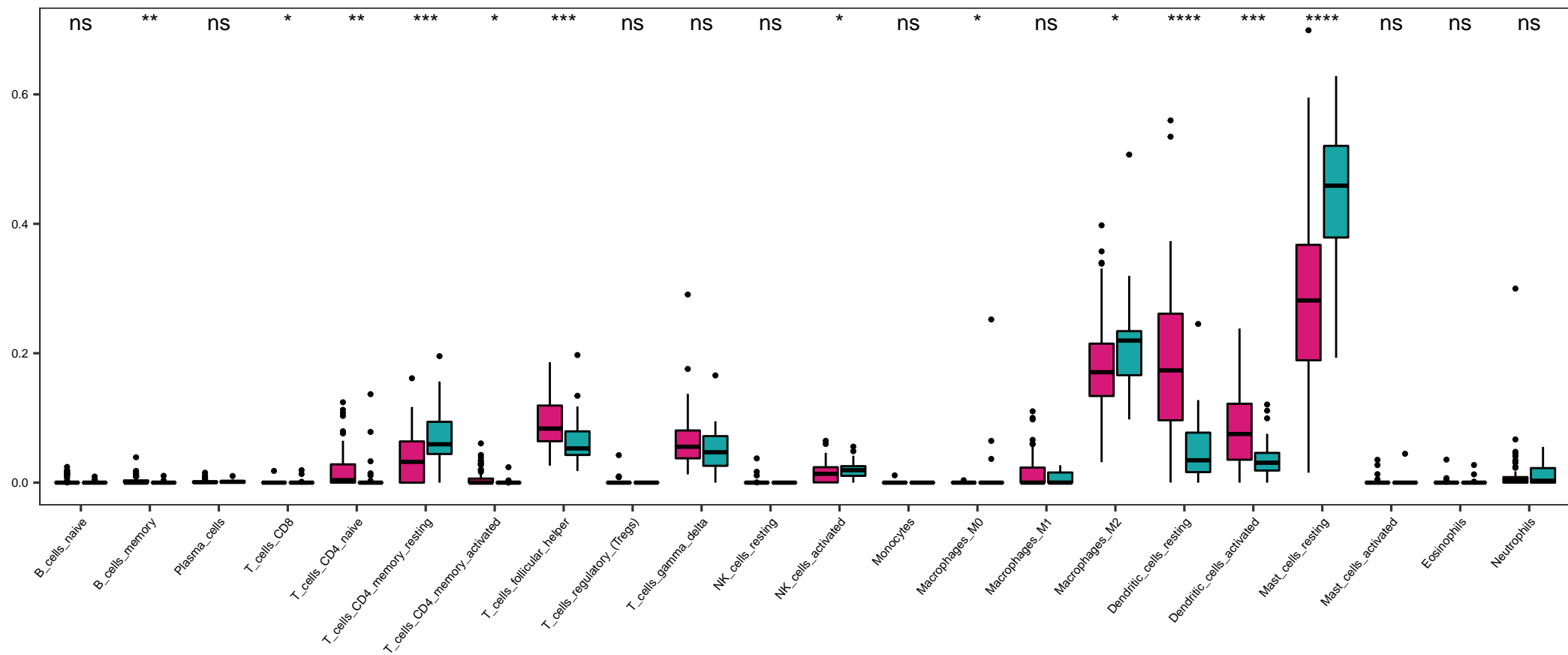

Supplement: Supplementary file 3 [file DataSheet1.pdf]

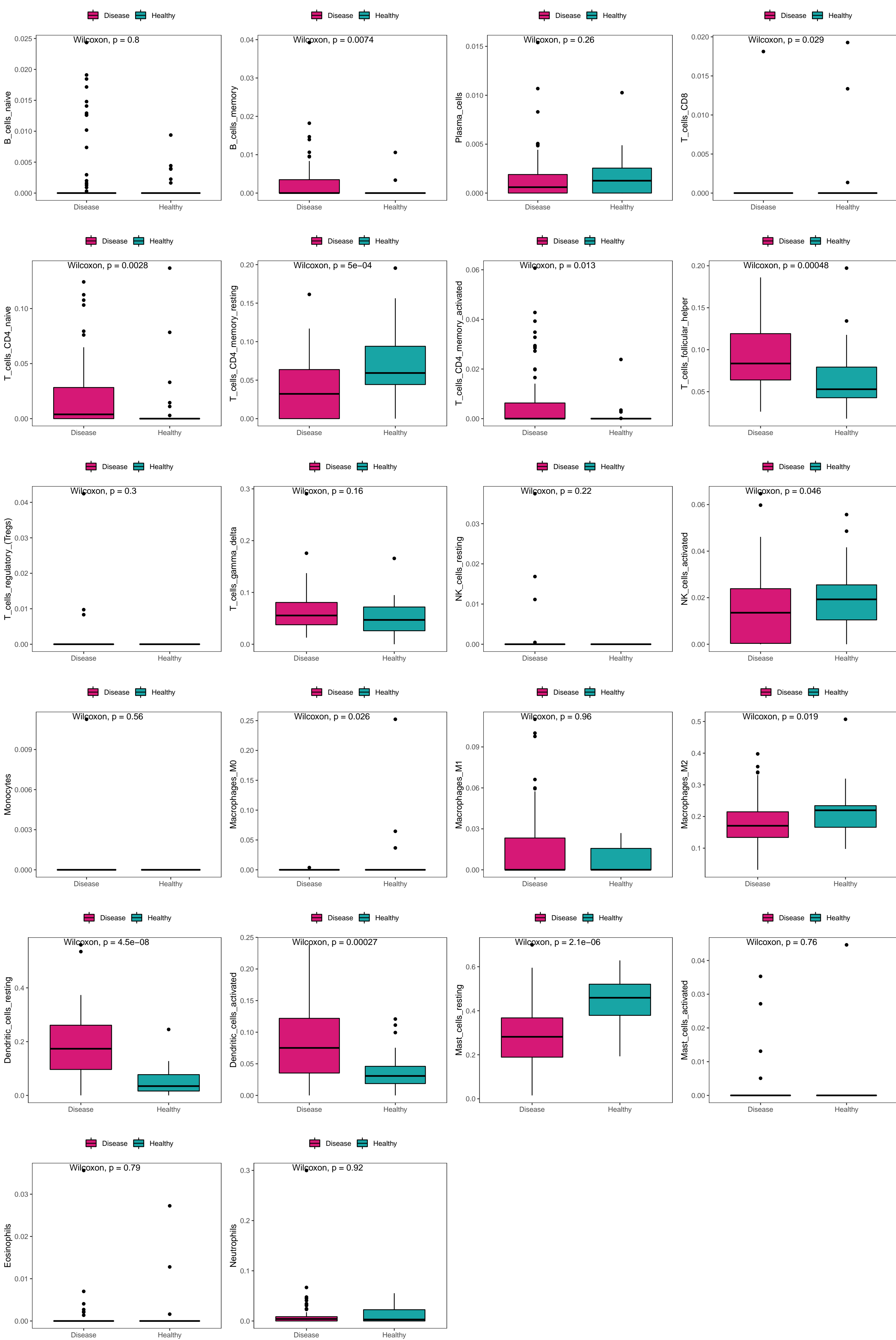

Supplement: Supplementary file 4 [file DataSheet2.pdf]

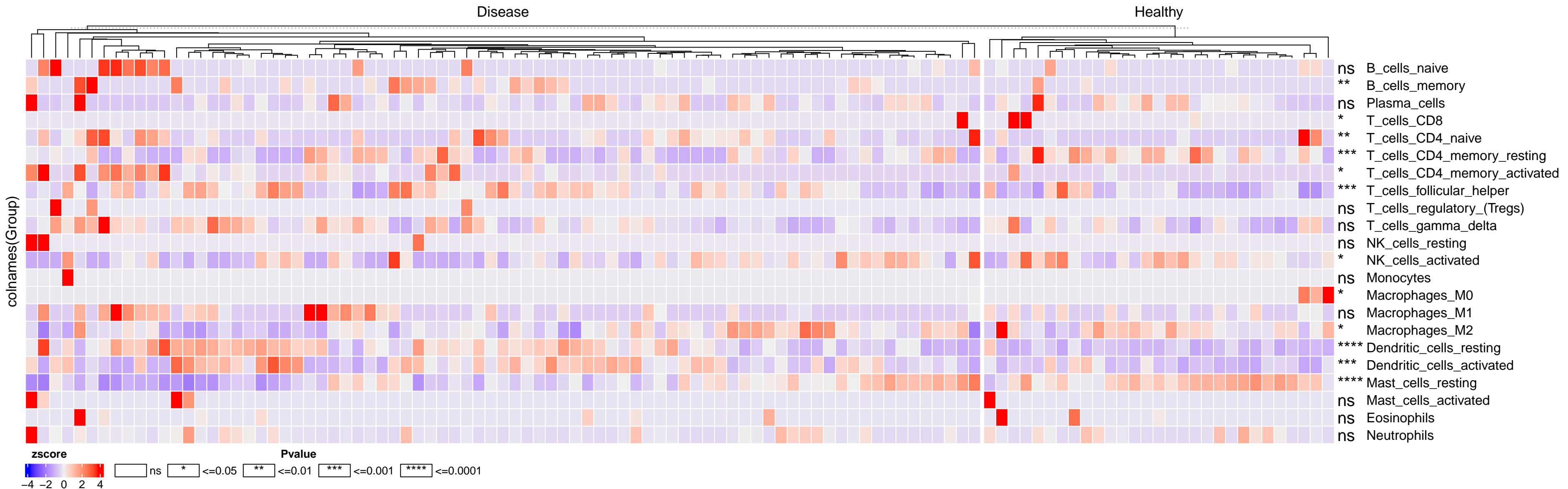

Supplement: Supplementary file 5 [file DataSheet3.pdf]

Disease Healthy

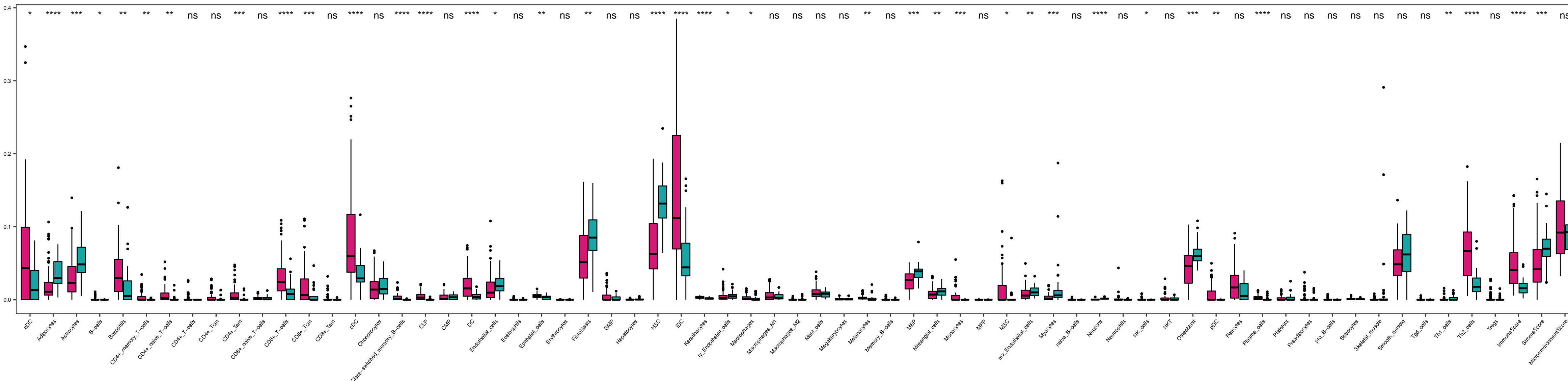

Supplement: Supplementary file 6 [file DataSheet4.pdf]

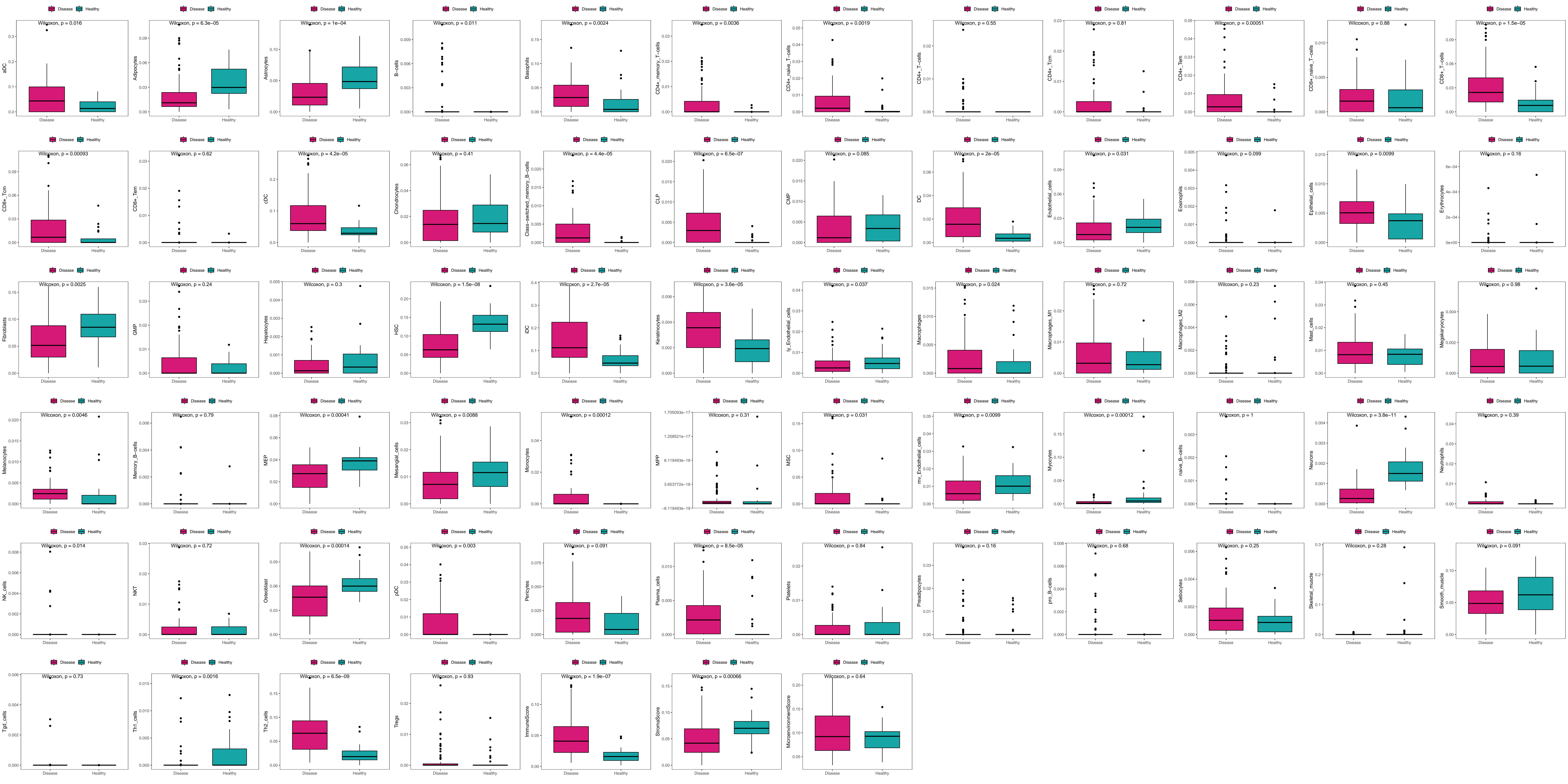

Supplement: Supplementary file 7 [file DataSheet5.pdf]

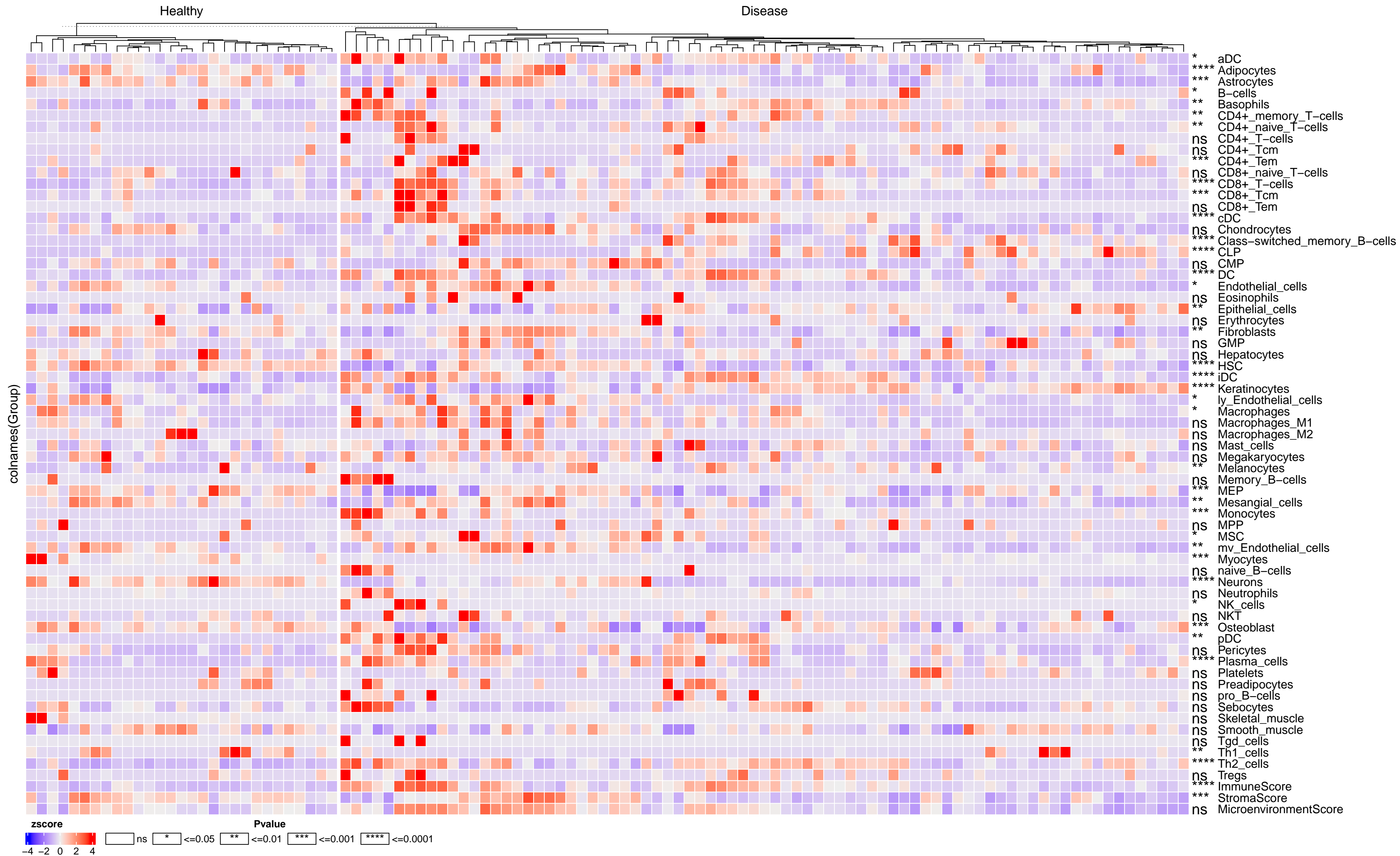

Supplement: Supplementary file 8 [file DataSheet6.pdf]
