# Supplementary material for: Identification and analysis of cuproptosis associated molecular clusters and immunological profiles in atopic dermatitis
Source: Front Immunol. 2025 Jun 27;16:1545457. doi: 10.3389/fimmu.2025.1545457 (PMC12245679; doi:10.3389/fimmu.2025.1545457)

Figure S1. The ssGSEA results using the GSE65832 bulk RNA-seq dataset (AD vs. healthy controls)

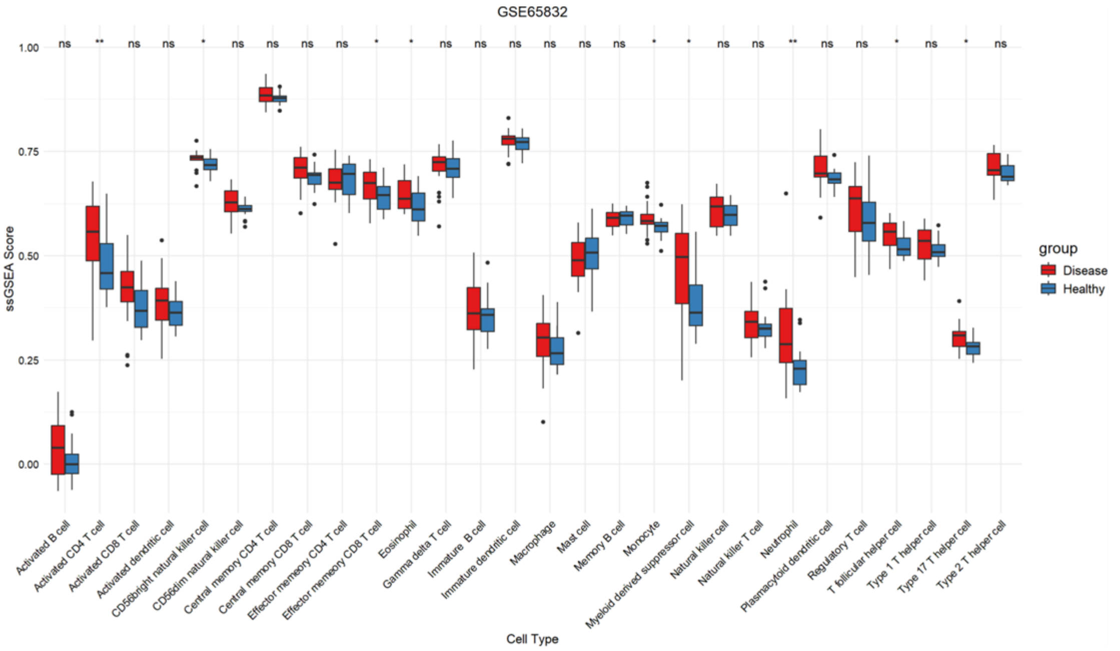

Figure S2. (**A**) UMAP plots of scRNA-seq data (GSE269981) showing immune cell clusters (T cells, B cells, neutrophils, NK cells) in AD lesional vs. healthy skin. (**B**) Bar plots quantifying immune cell frequencies. AD lesions exhibited higher CD4 T cells, B cells, neutrophils and dendritic cells compared to healthy controls.

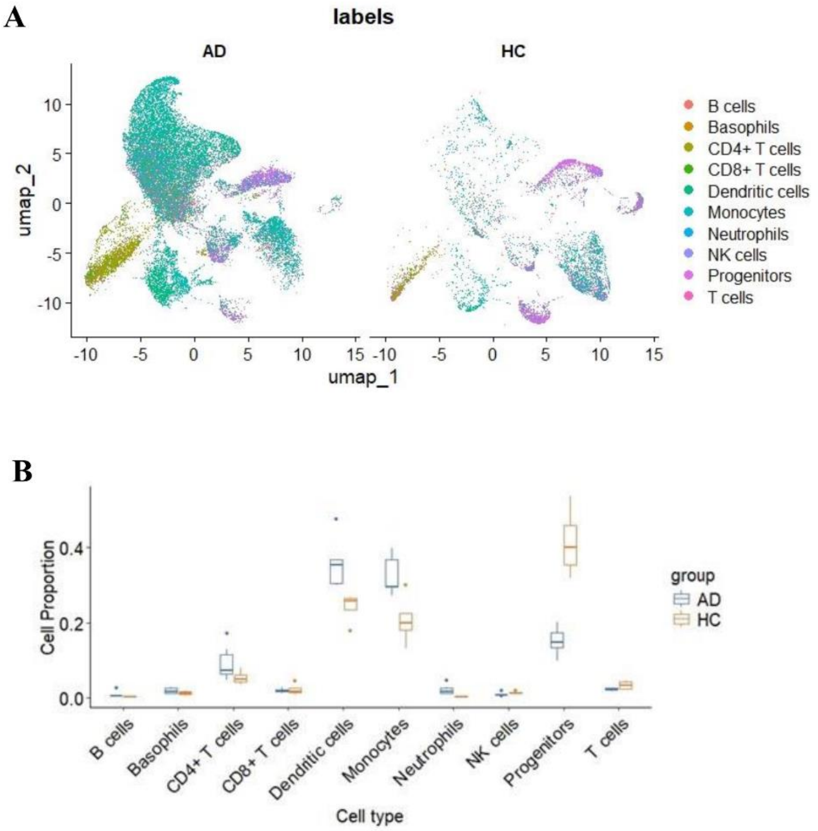

Supplement: Supplementary file 9 [file DataSheet7.pdf]
